# What's New from the British Journal of Surgery

**Published:** 1992-03

**Authors:** J. R. Farndon

**Affiliations:** Editor of the British Journal of Surgery & Chairman of the Editorial Board — West of England Medical Journal


### What's New from The British Journal of Surgery

**Digest prepared by: J. R. Farndon, BSc, MD, FRCS**
Editor of the British Journal of Surgery &
Chairman of the Editorial Board —
West of England Medical Journal

This digest gives areas of progress made in clinical and laboratory work relating to surgical disease. The digest is broken down into specialty areas so that you may wish to select only those of interest to your own practice.

### VASCULAR SURGERY

In January 1991 the winter meeting of the Surgical Research Society was held at St. Bartholomew's Hospital in London. On the first day a Symposium was held entitled "Shedding Light on Lasers". A review of this subject was published by Murray et al.[1] Different types of laser are described and the nature of various delivery devices. The physics and biology of laser tissue interaction depends upon the output wavelength and energy and upon the light absorption properties of the target tissues. Specific applications of laser technology in surgery include the treatment of carcinoma of the oesophagus and gastric cardia where laser vaporisation is best applied to exophytic lesions protruding into the lumen. Since one third of patients with rectosigmoid malignancy are incurable due to advanced locoregional or distant disease, laser palliation may be an ideal treatment to improve local problems of bleeding, discharge, tenesmus and obstruction with minimal morbidity and mortality. Laser tissue destruction may be effectively targeted using a chemical with a specific light wavelength. Haematoporphyrin derivative and dihaematoporphyrin ether (photofrin II) are the agents which have been used most clinically. Newer agents such as aluminum sulphonated phthalocyanine are being explored. These agents are preferentially retained by malignant tissues especially within the stroma. Photochemical reactions and tissue destruction are then specifically targeted by laser irradiation. A description of the use of lasers to achieve arterial recanalization completes this review article.

The same issue of the Journal carries an article by the same group describing laser assisted angioplasty as treatment for arterial occlusion in the lower limb.[2] A flashlamp-pumped pulse dye laser operating at 480 or 504nm was coupled to an integral ball-tipped optical fibre to recanalize occluded lower limb arteries. All channels created by the laser were augmented by balloon dilatation. Seventy-eight limbs in 71 patients were treated. The median occlusion length was 18cm and a technical success was achieved in 58 limbs (74%) with clinical success in 46 (59%). Greater success was achieved in shorter segment occlusions. Those with discrete arterial calcification had a lower technical success rate than those without calcification. Re-occlusion occured during follow-up; with a cumulative patency of 67% at six months, decaying to 45% at 12 months. It was possible to avoid bypass surgery or amputation in 46 treated limbs.

The December 1991 issue of the Journal contains three significant contributions describing clinical practice in patients with arterial disease. Hickey et al[3] describe an aggressive approach for arterial reconstruction in those with critical lower limb ischaemia. Patients were not excluded from limb salvage on the basis of poor run-off on preoperative angiography. Femorocrural bypass to a single calf vessel was required in 73% of the patients. The 30-day cumulative mortality rate was 7% rising to 41% by five years but cumulative graft patency at 30 days, one year, two years and five years was 96%, 85%, 84% and 82% respectively and was independent of the level of reconstruction. In situ long saphenous vein was the conduit of choice.

In a second paper in the same issue the authors show that failed femorocrural reconstruction did not prejudice subsequent amputation level.[4] Five hundred consecutive patients with limb threatening acute or critical ischaemia were studied and vascular reconstruction was attempted unless the patient had insufficient viable tissue to permit weight bearing or there was complete absence of run-off vessels in the calf. Fifty patients underwent primary amputation, 450 patients underwent vascular reconstruction and 265 of these had a femorocrural bypass. Sixty secondry amputations were required following failed femorocrural bypass. The below knee to above knee amputation ratio was two in the primary amputation group and 1.1 in the secondary amputation group. It was felt that an unselective policy of vascular reconstruction for critical ischaemia did not lead to a higher proportion of above knee amputations.

Embolic arterial lower limb occlusion presents as a more serious emergency for the vascular surgeon. Ljungman et al examined the risk factors for early lower limb loss after embolectomy for acute arterial occlusion.[5] The authors show that the amputation risk was increased in those patients who had undergone two or more myocardial infarctions, had chronic ischaemia, a long duration of symptoms or postoperative heart failure. Reduced risks were found in those with acute myocardial infarction and those on postoperative anticoagulant treatment with warfarin.

The Oxford group of vascular surgeons analysed the reoperation rate after major vascular surgery in a four year prospective analysis.[6] The authors demonstrate that patients undergoing major vascular surgery have a high reoperation rate (12%). Forty-nine per cent of the reoperations were attempts at revascularization, 32% were amputations and surgery for bleeding accounted for 13% of reoperations. The 30-day mortality rate for patients undergoing one reoperation was 13% and this figure rose to 35% if more than one reoperation was required. The authors rightly point to the significance of this data with regard to patient counselling and informed consent.

### HEPATOBILIARY DISEASE

Over the last few years there have been enormous advances in the treatment of hepatobiliary disease. We have now seen several junior residents who have become adept at removing the gallbladder laparoscopically when they have never carried out an open cholecystectomy!

This quarter's issues of the Journal have carried the usual complement of papers covering sugery of the biliary tree. One group in Dublin have a longstanding interest in surgery of the biliary tree and describe a new procedure for the treatment of high-risk patients with symptomatic gallstones.[7] Those aged over 60 with symptomatic stones who were at high operative risk underwent cholecystotomy under local anaesthesia through a 3cm incision. Stones were removed and clearance demonstrated endoscopically and by tube cholecystography. This procedure was successfully carried out in 24 of 26 patients. Four patients were shown to have common bile duct stones on cholecystography and these were treated successfully by endoscopic sphincterotomy. All treated patients were symptom-free at a mean follow-up of 36 weeks with no recurrent stones demonstrated by ultrasonography.

The group carried their studies further by demonstrating that cystic duct obliteration and gallbladder mucosal destruction was a feasible alternative to cholecystectomy.[8] This was an experimental study in mongrel dogs. A combination of duct electrocoagulation and delayed tetracycline installation at 14 days produced complete destruction of all gallbladder epithelium and an effective chemical cholecystectomy.

Subtotal cholecystectomy, we are reminded, is still a safe, straightforward and definitive operation in patients in whom standard cholecystectomy might be dangerous. Cottier et al[9] suggest that this is a more attractive proposition than cholecystostomy. Watch the debate!

We are reminded, however, that injury to our patient can still occur during biliary surgery whether open or laparoscopic. We need to audit very carefully the morbidity and mortality of new surgical procedures.

Whiston et al describe the development of a tension pneumothorax during laparoscopic cholecystectomy[10] and in December's leading article James Garden reviews 'Iatrogenic injury to the bile duct'[11] and places the issues with regard to mechanisms of injury and repair in current context.

If the gallstones do not cause acute cholecystitis they may cause acute pancreatitis. London et al[12] carried out a prospective study of rapid-bolus contrast-enhanced dynamic computed tomography as a means of evaluating pancreatic necrosis in severe pancreatitis. CT necrosis occurred significantly more frequently in patients with clinically severe (ten of 12) compared with mild (ten of 20) pancreatitis. Seven of 17 (41%) patients with CT necrosis developed clinically mild pancreatitis and six of 10 (60%) patients with clinically severe panceatitits and CT necrosis recovered with conservative management. The site and extent of CT necrosis did not correlate with disease severity. The authors conclude that the finding of CT necrosis is not in itself an indication for operative intervention. Rapid-bolus contrast-enhanced dynamic CT scanning facilitates the planning and execution of surgical therapy.

### COLORECTAL SURGERY

The Journal carries interesting papers for those involved in colorectal surgery. A surgical workshop by Chuah and Kelly[13] shows how to retrieve the polyp which has been removed colonoscopically and is always lost as the colonoscope is withdrawn from the anus. Using the simple expedient of threading the colonoscope through a proctoscope at the start of the procedure, those infuriating, exasperating lost polyps are guaranteed to be successfully retrieved in the future!

If you have to proceed to abdominoperineal excision of the rectum Dr. Campos and his colleagues from Murcia in Spain compared three methods of dealing with the perineal wound and pre-sacral space.[14] One hundred and two patients were randomized to have the perineal wound packed after suture of the pelvic peritoneum, suturing of the pelvic peritoneum and perineal wound leaving two drains through the perineum or thirdly, no suture of the pelvic peritoneum with primary closure of the perineal wound with drains through the abdomen for saline irrigation. The incidence of infection, primary healing rates, extraperineal complications and mean hospital stay were analysed. Primary healing and shortest hospital stay were best with the last method. The overall incidence of infection was highest with the second method. There were no differences between the methods with regard to extraperineal complications.

If you are suturing your large bowel anastomoses after resections for cancer you had better think again. Akyol et al[15] randomly allocated 294 patients undergoing potentially curative resection for colerectral cancer into two groups. One hundred and forty-two patients had sutured anastomoses and 152 had stapled anastomoses. Multiple regression analysis showed that the influence of the anastomotic technique on recurrence and mortality rate was independent of the tumour stage. Their results showed that stapling instruments were associated with a reduction in the incidence of recurrence and mortality rate by as much as 50%.

Two papers from Japan take us to new heights in the treatment of patients with colorectal carcinoma. Nakamura et al[16] show that in selected patients extensive and aggressive surgery for metastatic disease can be associated with apparently better survival. The group removed hepatic hilar lymph nodes and metastatic lesions from the liver, lung, adrenal gland and brain. The overall survival rate in 31 patients at five years was 45%. The outcome for six patients who underwent repeat hepatectomy was better than the nine patients with unresectable recurrence. Selected groups, perhaps, but nonetheless impressive results. The authors suggest that repeat hepatectomy and dissection of hepatic hilar lymph nodes improves prognosis in selected patients with hepatic metastases from colorectal cancer.

Kusunoki et al[17] show that in patients undergoing anoabdominal resection of the rectum with construction of a colonic J reservoir had a better quality of life than patients without a reservoir. Twenty-eight patients were studied and compared with eight patients without a reservoir at two years after surgery. Frequency of defaecation and daytime soiling were inversely correlated with the maximum tolerable volume of the colonic J pouch. Anal resting pressure, squeeze pressure, anal canal length and a positive inhibitor reflex were similar in both groups. Anal resting pressure, squeeze pressure and pouch distensibility correlated with the frequency of defaecation in the stable phase. The group suggests that pouch construction may improve the patient's quality of life in the adaptation phase.

### BENIGN ANORECTAL DISEASE

The Journal carries interesting papers covering aspects of benign anorectal disease.

The Birmingham group look at the need of a temporary loop ileostomy following restorative proctocolectomy.[18] A retrospective study compared the outcome of restorative proctocolectomy in those with and without a proximal loop ileostomy stoma. Although not randomly allocated to these groups those who had a loop ileostomy had a higher incidence of anastomotic leakage, pelvic abscess and postoperative fistula than those without a covering ileostomy. Intestinal obstruction occurred in 23% of those with an ileostomy compared with 6% in those without a stoma. The group suggests that omission of an ileostomy may be possible if the surgeon is suitably experienced, a technically good anastomosis is carried out and if the patient is fit and has not received steroids. They suggest that a prospective controlled study is needed to answer the question.

The St. Mark's group show that if you are trying to cure rectal prolapse by rectopexy, if you divide the lateral ligaments the patients will be constipated but the risk of recurrence will be less.[19] Patients were randomized prospectively to rectopexy with (n=14) or without (n=12) division of the lateral ligaments. Incontinence was improved in both groups of patients but division of the lateral ligaments increased the number of patients with constipation compared to those without division of the ligaments. This might relate to changes in the rectal electrical sensory threshold which was markedly increased after lateral ligament division. Prolapse recurred in a higher proportion of patients who did not undergo division of the lateral ligaments. You win on the swings but you lose on the roundabouts!

If you are left with a patient with severe constipation then don't forget the possibility of using antegrade continence enemas. Wheeler and Malone describe the use of the appendix in reconstructive surgery.[20] They argue against incidental appendicectomy. There are many other arguments against incidental appendicectomy but these authors describe the possible uses of the appendix in reconstructive surgery. The appendix can be a useful continence stoma facilitating catheterization of a denervated bladder or after urinary diversion. The review describes the use of the appendix to enable antegrade continence enema administration for children with chronic constipation or faecal incontinence. The appendix is reversed and brought onto the abdominal wall as a continent stoma, the distal end being implanted in a non-refluxing fashion into the caecum. The appendix then provides a continent catheterizable channel which is available for antegrade enema administration. This may allow dramatically improved bowel care in patients with incapacitating constipation or incontinence. It has enabled wheelchair-bound patients to take care of bowel function themselves and allow independent existence.

If you do not need further evidence to persuade you to stop carrying out anal dilatation, read no further. Speakman et al[21] provide convincing evidence of sphincter injury demonstrated by anal endosonography after anal dilatation in a selected group of 12 men presenting with faecal incontinence following anal dilatation. Eleven showed a disrupted internal sphincter on endosonography. Three patients showed defects in the external sphincter. Resting anal pressures were low and pudendal nerve latencies were normal. These findings demonstrate that the structural injury caused by anal dilatation is sphincter disruption.

**BREAST DISEASE**

It is not always possible to obtain mammograms before a lady is seen in the outpatient clinic and a fine needle biopsy carried out. Do the changes within the breast caused by the fine needle biopsy affect subsequent mammograms? Horobin et al[22] examine this question in 52 women who underwent mammography before and within five days of needle aspiration. These mammograms were examined by one radiologist who did not know the timing of the films. Significant differences were only seen in ten patients. In seven of these the difference was due to an aspirated cyst. In three patients the differences were unexpected and more significant but the radiological diagnosis was not altered. The authors felt that some shadows might be interpreted as falsely malignant and some small cancers might be obscured by haematoma formation. Patients accept mammography more readily before needle aspiration than afterwards because of increased discomfort. The authors suggest that mammography should be carried out before clinical examination and fine needle aspiration because of the risk of false interpretation and because of better patient acceptance. This new system of clinical practice is being prospectively evaluated by the authors.

If you thought the blood transfusion controversy was over then read another paper about the blood transfusion effect on recurrence and death after mastectomy for breast cancer. Eickhoff et al[23] from the Danish Breast Cancer Cooperative Group studied the effect of blood transfusion on 1 599 patients undergoing mastectomy for carcinoma of the breast. Two hundred and twenty-eight patients received a transfusion and the disease recurred in 110 (48%). This compared with recurrence in 647 of 1 371 non-transfused patients (47%). The five year recurrence-free survival rate was 0.54 in the transfused group and 0.60 in the non-transfused group. These differences were not significant. The transfused patients had a higher prevalence of risk ractors than the non-transfused patients. Cox's multiple regression analysis, however, confirmed that perioperative blood transfusion was of no significance. This Danish study did not add support to the hypothesis that perioperative blood transfusion promotes recurrence after surgery for breast cancer.

**REFERENCES**

1. Murray A., Mitchell D.C., Wood R.F.M. Lasers in Surgery. *Br. J. Surg.* 1992. **Vol 79:** 21-26.
2. Mitchell D.C., Murray A., Wood R.F.M., Grasty M., Smith R.E., Dacie J.E., Walters T.K., Cotton G. Laser-assisted angioplasty for arterial occlusion of the lower limb: initial results and follow-up. *Br. J. Surg.* 1992. **Vol. 79:** 81-85.
3. Hickey N.C., Thomson I.A., Shearman C.P., Simms M.H. Aggressive arterial reconstruction for critical lower limb ischaemia. *Br. J. Surg.* 1991: **Vol. 78:** 1476-1478.
4. Tsang G.M.K., Crowson M.C., Hickey N.C., Simms M.H. Failed femorocrural reconstruction does not prejudice amputation level. *Br. J. Surg.* 1991: **Vol. 78:** 1479-1481.
5. Ljungman C. Adami H-O, Bergqvist D., Sparen P., Bergstrom R. Risk factors for early lower limb loss after embolectomy for acute arterial occlusion: a population-based case-control study. *Br. J. Surg.* 1991: **Vol. 78:** 1482-1485.
6. Davies A.H., Pope I., Collin J., Morris P.J. Early reoperation after major vascular surgery: a four-year prospective analysis. *Br. J. Surg.* 1992: **Vol. 79:** 76-78.
7. Leahy A.L., Darzi A.W., Murchan P.M., O'Gorman S., Hamilton S. Tanner W.A., Keane F.B.V. A safe new procedure for high risk patients with symptomatic gallstones. *Br. J. Surg.* 1991: **Vol. 78:** 1319-1320.
8. Leahy A.L., McCollum P.T., O'Gorman S., Darzi A., Marks P., Kay E., Tanner W.A., Keane F.B.V. Cystic duct obliteration and gallbladder mucosal destruction: a feasible alternative to cholecystectomy. *Br. J. Surg.* 1991: **Vol. 78:** 1321-1324.
9. Cottier D.J., McKay C., Anderson J.R. Subtotal cholecystectomy. *Br. J. Surg.* 1991: **Vol. 78:** 1326-1328.
10. Whiston R.J., Eggers K.A., Morris R.W., Stamatakis J.D. Tension pneumothorax during laparoscopic cholecystectomy. *Br. J. Surg.* 1991: **Vol. 78:** 1325.
11. Garden O.J. Latrogenic injury to the bile duct. *Br. J. Surg.* 1991: **Vol. 78:** 1412-1413.
12. London N.J.M., Leese T., Lavelle J.M., Miles K., West K.P., Watkin D.F.L., Fossard D.P. Rapid-bolus contrast-enhanced dynamic computed tomography in acute pancreatitis: a prospective study. *Br. J. Surg.* 1991: **Vol. 78:** 1452-1456.
13. Chuah S.Y., Kelly M.J. Proctoscopic polyp delivery after colonoscopic polypectomy. *Br. J. Surg.* 1992: **Vol. 79:** 32.
14. Robles Campos R., Garcia Ayllon J., Parrilla Paricio P., Cifuentes Tebar J., Lujan Mompean J.A., Liron Ruiz R., Torralba Martinez J.A., Molina Martinez J. Management of the perineal wound following abdominoperineal resection: prospective study of three methods. *Br. J. Surg.* 1992: **Vol 79:** 29-31.
15. Akyol A.M., McGregor J.R., Galloway D.J., Murray G., George W.D. Recurrence of colorectal cancer after sutured and stapled large bowel anastomoses. *Br. J. Surg.* 1991: **Vol. 78:** 1297-1300.
16. Nakamura S., Yokoi Y., Suzuki S., Baba S., Muro H. Results of extensive surgery for liver metastases in colorectal carcinoma. *Br. J. Surg.* 1992: **Vol. 79:** 35-38.
17. Kusunoki M., Shoji Y., Yanagi H., Hatada T., Fujita S., Sakanoue Y., Yamamura T., Utsunomiya J. Fuction after anoabdominal rectal resection and colonic J pouch-anal anastomosis.
18. Hosie K.B. Grobler S.P. Keighley M.R.B. Temporary loop ileostomy following restorative proctocolectomy. *Br. J. Surg.* 1992: **Vol. 79:** 33-34.
19. Speakman C.T.M., Madden M.V., Nicholls R.J., Kamm M.A. Lateral ligament division during rectopexy causes constipation but prevents recurrence: results of a prospective randomized study. *Br. J. Surg.* 1991: **Vol. 78:** 1431-1433.
20. Wheeler R.A., Malone P.S. Use of the appendix in reconstructive surgery: a case against incidental appendicectomy. *Br. J. Surg.* 1991: **Vol. 78:** 1283-1285.
21. Speakman C.T.M., Burnett S.J.D., Kamm M.A., Bartram C.I. Sphincter injury after anal dilatation demonstrated by anal endosonography. *Br. J. Surg.* 1991: **Vol. 78:** 1429-1430.
22. Horobin J.M. Matthew B.M., Preece P.E., Thompson A.J. Effects of fine needle aspiration on subsequent mammograms. *Br. J. Surg.* 1992: **Vol. 79:** 52-54.
23. Eickhoff J.H., Andersen J., Laybourn C. Perioperative blood transfusion does not promote recurrence and death after mastectomy for breast cancer. *Br. J. Surg.* 1991: **Vol. 78:** 1358-1361.

